# Metabolomics in the Identification of Biomarkers of Asthma

**DOI:** 10.3390/metabo11060346

**Published:** 2021-05-29

**Authors:** Alma Villaseñor, Ibon Eguiluz-Gracia, André Moreira, Craig E. Wheelock, María M Escribese

**Affiliations:** 1 Department of Basic Medical Sciences, Facultad de Medicina, Institute of Applied Molecular Medicine (IMMA), Universidad San Pablo CEU, CEU Universities, 28660 Madrid, Spain; alma.villasenor@ceu.es; 2 Centre for Metabolomics and Bioanalysis (CEMBIO), Department of Chemistry and Biochemistry, Facultad de Farmacia, Universidad San Pablo-CEU, CEU Universities, Urbanización Montepríncipe, Boadilla del Monte, 28660 Madrid, Spain; 3 Allergy Group, Instituto de Investigacion Biomedica de Malaga (IBIMA) and ARADyAL, 29009 Malaga, Spain; iboneguiluz@gmail.com; 4 Allergy Unit, Hospital Regional Universitario de Malaga, 29009 Malaga, Spain; 5 EPI Unit, Instituto de Saúde Pública, Universidade do Porto, 4050-600 Porto, Portugal; andremoreira@med.up.pt; 6 Serviço de Imunologia Básica e Clínica, Departamento de Patologia, Faculdade de Medicina da Universidade do Porto, 4200-319 Porto, Portugal; 7 Serviço de Imunoalergologia, Centro Hospitalar São João EPE, 4200-319 Porto, Portugal; 8 Division of Physiological Chemistry 2, Department of Medical Biochemistry and Biophysics, Karolinska Institutet, Biomedicum Quartier 9A, SE-171-77 Stockholm, Sweden; craig.wheelock@ki.se; 9 Department of Respiratory Medicine and Allergy, Karolinska University Hospital, SE-171-77 Stockholm, Sweden; 10 Gunma University Initiative for Advanced Research (GIAR), Gunma University, 3-39-22 Showa-machi, Maebashi 371-8511, Gunma, Japan

## 1. Asthma

Asthma is a major non-communicable disease characterized by recurrent attacks of breathlessness and wheezing. According to World Health Organization (WHO), it is estimated that more than 339 million people had asthma globally in 2016. Indeed, there were 417,918 deaths, due to asthma at the global level.

The concept of asthma as a single entity has now evolved into a much more complex biological network of distinct and interrelating inflammatory pathways. Asthma is considered a multifactorial, chronic syndrome, which varies over time and involves genetic and environmental interactions. It causes reversible airway obstruction through spasm, inflammation, and hypersecretion associated with airway hyperresponsiveness (AHR), infiltration of immune cells into the airway submucosa, and airway epithelial remodeling [1]. This heterogeneity reflects the underlying mechanisms (endotypes) and variable clinical presentations (phenotypes) [2]. The inflammatory phenotypes identified to date include eosinophilic or T2 or T2-high asthma, and non-eosinophilic, non-T2 or T2-low asthma, which comprises, in turn, the neutrophilic and paucigranulocytic phenotypes. Another level of complexity resides because even with similar clinical symptoms, patients may respond differently to the same therapeutic interventions [3]. Stratification of patients for treatment is limited to the measurement of blood or sputum eosinophils, fractional exhaled nitric oxide (FENO), or protein markers in the blood, such as total IgE or periostin that do not provide consistent information [4]. There is accordingly an unmet need to identify new predictive biomarkers to improve the stratification of patients by pathobiological mechanisms and to aid in the selection of treatments.

Additionally, it is also relevant to reveal that the increased prevalence of asthma can be partly accounted for by the profound changes in the exposome related to Western lifestyle (high level of urbanization, isolation of households, workplaces, and schools where subjects spend most of their time, etc.). The Western lifestyle is also associated with a rise in outdoor pollution and increased exposure to indoor pollution (e.g., tobacco smoke) and indoor allergens (e.g., animal dander and mites) [5,6]. Indeed, house dust mites (HDM) are the most prevalent allergens associated with asthma and rhinitis worldwide. It is likely that different triggers might also affect the inflammatory response [1].

Asthma in children remains a significant public health challenge affecting 5–20% of children in Europe, associated with increased morbidity and societal healthcare costs. Identifying early diagnostic biomarkers and improving the monitoring of airway dysfunction and inflammatory through non-invasive methods are key goals in successful pediatric asthma management [7]. 

In adults, asthma is the most common chronic airway inflammation with multiple phenotypes caused by complicated interactions of genetic, epigenetic, and environmental factors. Disability and global impairment of quality of life result from a complex interplay of these factors in adults. Identification of biomarkers and new causative factors may possibly lead to more personalized and precise pathway-specific approaches for asthma diagnosis and treatment.

There is an urgent need to investigate and generate new knowledge regarding the inflammatory pathways involved in the trigger and progression of this disease, both in vitro and in vivo models that enable us to: (1) Understand asthmatic endotypes and phenotypes with special focus in severe asthmatic patients, both adults and pediatrics; and (2) develop better prevention and intervention strategies. 

## 2. Metabolomics

Metabolomics focuses on studying compounds defined as metabolites that encompass the metabolism of a living organism/tissue/cell [8]. This is a valuable tool for biomarker discovery, predicting response to therapy and potential pathogenic pathways for various complex diseases, such as asthma. Characterization of the metabolic profile can capture metabolic alterations in asthma and has revealed alterations in cellular energy, amino acid, oxidative stress, fatty acid, sphingolipid, and phospholipid metabolism [9]. Complementary, lipid metabolism has been emphasized to be related to lung dysfunction in mild-to-moderate asthma, as well as asthma in obese patients [10]. Thus, metabolomics is an emerging approach that may provide more insight into asthma pathophysiological mechanisms, enable the identification of early biomarkers and targeted personalized therapies, thus reducing the disease burden and societal cost.

Metabolomics can be performed following two different approaches: Untargeted and targeted metabolomics. In the untargeted analysis, the aim is to detect as many metabolites as possible in a single run analysis for each sample from the study, and to identify those with a statistical difference between the experimental groups. In the case of the targeted analysis, specific metabolites selected based upon previous knowledge are analyzed and frequently quantified. The untargeted approach is the first step in an exploratory study to find promising metabolic changes, which later on, could help to better understand the molecular mechanisms in the pathology. This can result in the identification of biomarkers, which after validation can be used in the clinic for diagnosis or prognosis of disease. 

Due to the diverse physicochemical properties of metabolites, metabolomics needs sophisticated analytical techniques, which permit the characterization of the metabolites. As such, metabolomics is usually performed by mass spectrometry (generally coupled to a separation technique, such as gas and liquid chromatography, and capillary electrophoresis, GC-MS, LC-MS, and CE-MS, respectively) or nuclear magnetic resonance spectroscopy (NMR), which are used in search of biomarkers of different asthma endotypes, as well as response to treatment. Metabolic changes on the cellular level of the host cells and microbiota are reflected on the systemic level in the circulation at the blood, urine, induced sputum, bronchoalveolar lavage (BAL), and exhaled air. Analysis of all these biological matrices in the contexts of animal and human models will help understand the mechanisms in this complex pathology. In addition, animal and human studies point to a prominent role of the gut microbiome in asthma development, relevant metabolomic mechanisms behind this association are beginning to be elucidated [11].

As an example, a recent study in childhood asthma using metabolomics suggests the existence of an endotype with early onset and increased airway resistance that is characterized by reduced sphingolipid concentrations, which are associated with 17q21 genetic variants and expression of the serine palmitoyl-CoA transferase (SPT) enzyme [12].

**Figure 1:**
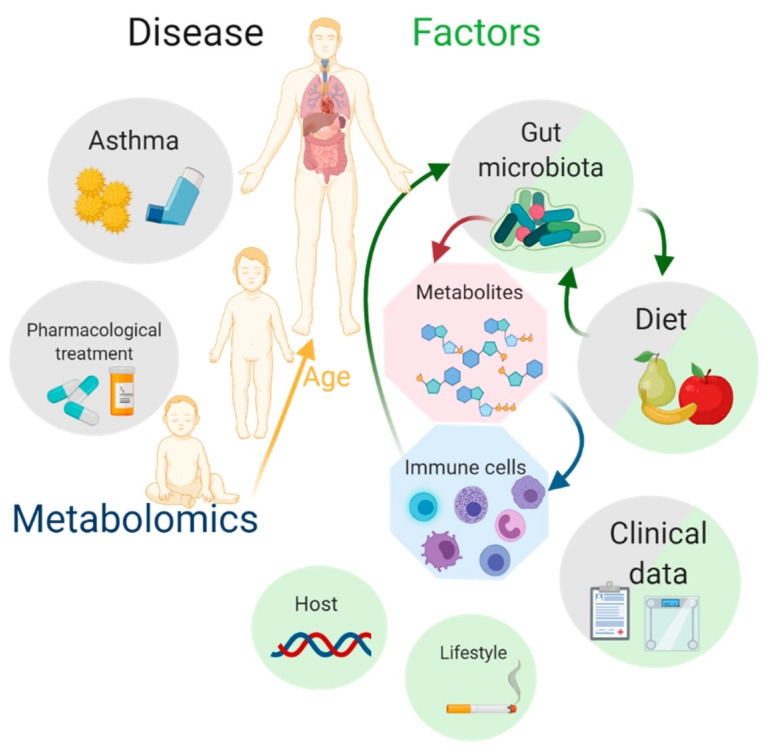
Metabolomics in the quest of asthma biomarkers. Some of the main factors that affect metabolites are diet, lifestyle, genetics, clinical parameters, and gut microbiota. There is a close relationship between intestinal microbiota, serum metabolites, and immune cells. All these factors are also connected with asthma, their pharmacology treatment, and age.

The purpose of this Special Issue is to provide evidence of the utility of metabolomics in asthma (pediatric and adult), and to show their potential application in clinical practice. Moreover, current challenges in integrating metabolomics in asthma management are also critically presented (Figure 1).
